# Assessment of the CHA_2_DS_2_-VASc Score for the Prediction of Death in Elderly Patients With Coronary Artery Disease and Atrial Fibrillation

**DOI:** 10.3389/fcvm.2021.805234

**Published:** 2021-12-24

**Authors:** Yangxun Wu, Guanyun Wang, Lisha Dong, Liu'an Qin, Jian Li, Hengming Yan, Wenjie Guo, Xiaodong Feng, Yuting Zou, Ziqian Wang, Rina Du, Yuxiao Zhang, Jing Ma, Tong Yin

**Affiliations:** ^1^Institute of Geriatrics, National Clinical Research Center for Geriatric Diseases, 2nd Medical Center, Medical School of Chinese People's Liberation Army and Chinese People's Liberation Army General Hospital, Beijing, China; ^2^Department of Cardiology, 1st Medical Center, Medical School of Chinese PLA and Chinese PLA General Hospital, Beijing, China

**Keywords:** elderly, atrial fibrillation, coronary artery disease, death, CHA_2_DS_2_-VASc score

## Abstract

**Purpose:** Coronary artery disease (CAD) and atrial fibrillation (AF) often coexist and lead to a much higher risk of mortality in the elderly population. The aim of this study was to investigate whether the CHA_2_DS_2_-VASc score could predict the risk of death in elderly patients with CAD and AF.

**Methods:** Hospitalized patients aged ≥65 years with a diagnosis of CAD and AF were recruited consecutively. Patients were divided into 5 groups according to the CHA_2_DS_2_-VASc score (≤2, =3, =4, =5, and ≥6). At least a 1-year follow-up was carried out for the assessment of all-cause death.

**Results:** A total of 1,579 eligible patients were recruited, with 582 all-cause deaths (6.86 per 100 patient-years) occurring during a follow-up of at least 1 year. With the increase in the CHA_2_DS_2_-VASc score, the 1-year and 5-year survival rate decreased (96.4% vs. 95.7% vs. 94.0% vs. 86.5% vs. 85.7%, respectively, *P* < 0.001; 78.4% vs. 68.9% vs. 64.6% vs. 55.5% vs. 50.0%, respectively, *P* < 0.001). Compared with the patients with CHA_2_DS_2_-VASc score <5, for patients with CHA_2_DS_2_-VASc score ≥5, the adjusted hazard ratio for death was 1.78 (95% CI: 1.45–2.18, *P* < 0.001). The predictive values of the CHA_2_DS_2_-VASc score ≥5 for in-hospital (C-index = 0.66, 95% CI: 0.62–0.69, *P* < 0.001), 1-year (C-index = 0.65, 95% CI: 0.63–0.67, *P* < 0.001) and 5-year (C-index = 0.60, 95% CI: 0.59–0.61, *P* < 0.001) death were in comparable.

**Conclusion:** In elderly patients with concomitant CAD and AF, the CHA_2_DS_2_-VASc score can be used to predict death with moderate accuracy.

## Introduction

Coronary artery disease (CAD) is the most common cardiovascular disease, while atrial fibrillation (AF) is the most common cardiac arrhythmia (1). The prevalence of both CAD and AF increases monotonically with age (2, 3). CAD and AF often coexist and interact with each other (4). CAD is a leading cause of morbidity and mortality in elderly adults (5). Elderly patients are more likely than their younger counterparts to present with comorbidities (6–9), contributing to worse outcomes. Patients with AF are relatively older and have higher risk of stroke, which may contribute to increased mortality (10–16). Furthermore, AF is a well-established marker of poor short- and long-term prognosis in patients with acute myocardial infarction (AMI) (11, 12, 17, 18) and is associated with a large increase in overall mortality (15, 19). Therefore, the coexistence of CAD and AF might lead to a much higher risk of mortality in the elderly population.

The CHA_2_DS_2_-VASc score [congestive heart failure, hypertension, age ≥75 years (doubled), diabetes, stroke/transient ischemic attack/thromboembolism (doubled), vascular disease (prior myocardial infarction, peripheral artery disease, or aortic plaque), age 65–74 years, sex category (female)] has been used for the assessment of thromboembolic (TE) risk and the guidance of antithrombotic treatment in patients with AF (20). In addition, this simple and well-established scoring system has been shown to predict the risk for other conditions beyond its original designations (20–26).

In the presence of comorbidities of CAD and AF, advancing age further elevates the risk of TE complications and death. Evaluating the risk of death from TE in an elderly population with CAD and AF is important, because a competing-risk setting taking careful consideration of the interplay between the mortality of elderly individuals with CAD and AF, and mortality of ischemic stroke/TE is needed to provide meaningful risk assessments. However, how to assess the relationship between the mortality of ischemic stroke/TE and the high mortality of the elderly population is still unclear. Therefore, we aimed to evaluate whether the CHA_2_DS_2_-VASc score can predict the risk of death in elderly CAD and AF patients and be used as an indicator of treatment and prognosis.

## Methods

### Patients

From January 2010 to December 2017, patients aged ≥65 years with a diagnosis of both CAD and AF who were hospitalized in the Department of Cardiology, Chinese PLA General Hospital, were recruited consecutively. This study complied with the Declaration of Helsinki and was approved by the institutional ethics committee of the General Hospital of the People's Liberation Army. CAD was defined as stable coronary artery disease (SCAD, including stable angina, previous myocardial infarction and ischemic cardiomyopathy) and acute coronary syndrome (ACS, including unstable angina and acute myocardial infarction). AF was defined as an irregular rhythm recorded in a standard 12-lead electrocardiogram, including discrete P waves and their replacement with irregular chaotic oscillatory atrial activity (F waves) in the setting of irregular QRS complexes. Body mass index (BMI) was categorized according to the distribution of BMI among the patients and the WHO criteria (27). In line with the epidemiological evidence, a BMI from 22 to <25 kg/m^2^ was used as the reference group. On defining the different classification of HF, we summarized the left ventricular ejection fraction (LVEF) data of the included CAD and AF patients. Patients with HF were stratified into 3 groups according to the criteria that LVEF <40% represents heart failure with reduced ejection fraction (HFrEF), LVEF ≥50% represents heart failure with preserved ejection fraction (HFpEF), and LVEF ranges from 40 to 50 represents heart failure with mid-range ejection fraction (HFmrEF).

### Data Collection, Follow-Up and Death Definitions

Baseline demographics and clinical characteristics in the hospital were extracted from the electronic health records system. The components of the CHA_2_DS_2_-VASc score were collected to retrospectively assess the risk of mortality. The CHA_2_DS_2_-VASc score was calculated as congestive heart failure (1 point), aged 65–74 years (1 point), hypertension (1 point), diabetes (1 point), vascular disease (prior myocardial infarction, peripheral artery disease, or aortic plaque; 1 point), female sex (1 point), aged 75 years or older (2 points) and stroke/transient ischemic attack/thromboembolism (2 points) (28, 29).

Participants were followed-up until Dec 31st, 2019. The follow-up protocol included a combination of hospital medical record reviews, telephone contacts with patients or family members and death certificate reviews. All deaths were independently adjudicated in a blinded manner by 2 members of the event adjudication committee. All-cause death was classified using the tenth revision of the International Classification of Disease and confirmed through death certificates using personal identity card numbers. Cardiac death was defined as death attributable to fatal myocardial infarction, sudden cardiac death or stroke. Apart from cardiac death, non-cardiac death included deaths from malignancies, infections, respiratory, trauma/accidental or other non-vascular deaths. If the cause of death could not be determined from the available evidence, death was classified as undetermined.

### Statistical Analysis

The patients were divided into CHA_2_DS_2_-VASc score groups (≤2, =3, =4, =5, and ≥6) according to whether they had died by the end of follow-up. Baseline demographic and clinical characteristics were summarized using medians and interquartile ranges (IQRs) for continuous measures and percentages for categorical measures. The comparison of the data was performed using the chi-square test for categorical variables and Mann–Whitney *U*-test for continuous variables. Univariate and multivariate Cox regression models were used to explore the risk factors associated with mortality. According to the CHA_2_DS_2_-VASc score, Kaplan–Meier curves with the log-rank test were used to compare survival. The calibration of the CHA_2_DS_2_-VASc score was assessed with the Hosmer-Lemeshow goodness-of-fit test (HL), which may determine the degree of agreement between the observed event rate and the predicted one over a series of scores. A significant value of *P* < 0.05 represents a lack of fit. The concordance index (C-index) was conducted to determine the discrimination of and the diagnostic value of the CHA_2_DS_2_-VASc score for death. All analysis were performed with the R 4.0.1 Statistical Package (the R foundation for Statistical Computing, Vienna, Austria) and SPSS v.24.0 (Statistical Package for Social Science; IBM, Chicago, IL, USA).

## Results

### Baseline Characteristics

**Figure 1:**
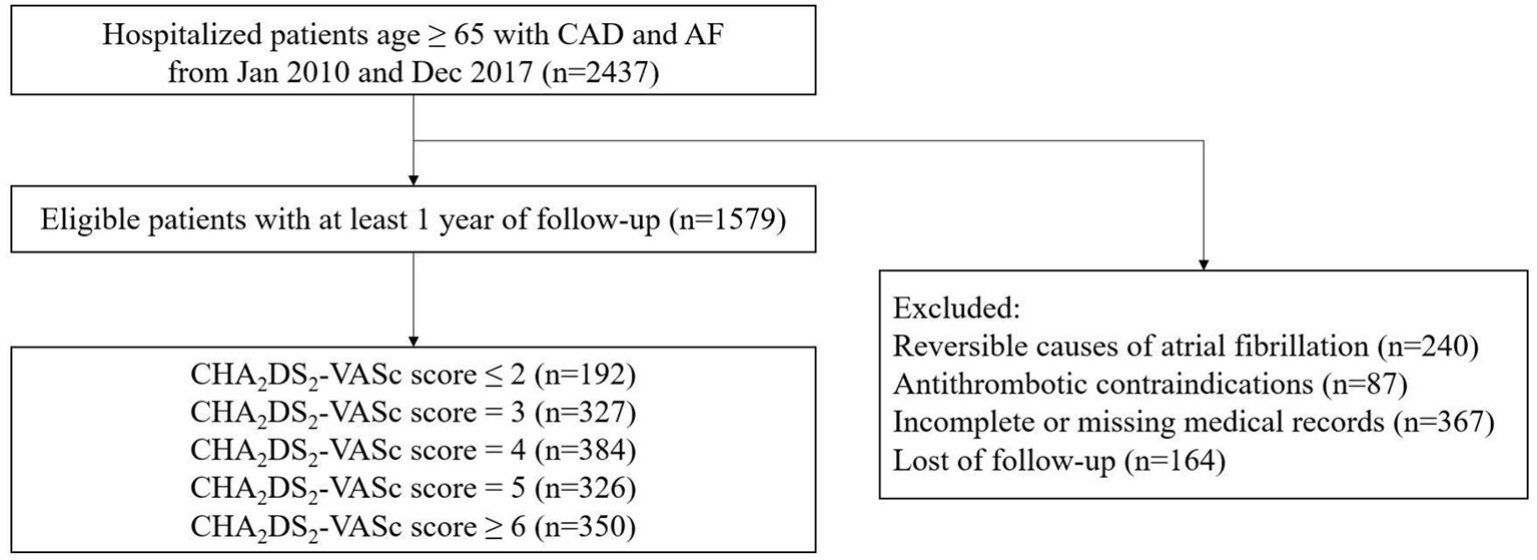
Flow diagram of the study.

Follow-up data were available for 1,579 patients (with a total of 1,579 patients for 1-year and 910 for 5-year follow-up). The baseline characteristics according to the CHA_2_DS_2_-VASc score are shown in Table 1. The mean CHA_2_DS_2_-VASc score was 4.3 ± 1.6 (median 4.0, interquartile range 3.0–5.0). We divided the cohort into five quintiles based on the CHA_2_DS_2_-VASc score: ≤2 (*n* = 192), 3 (*n* = 327), 4 (*n* = 384), 5 (*n* = 326), and ≥6 (*n* = 350) (Figure 1). Patients with a higher CHA_2_DS_2_-VASc score were more likely to be women, older and with cardiovascular diseases, such as AMI and heart failure (HF). The proportion of patients with HF was 38.3% (602/1579) in the whole study. Among the patients with HF, LVEF data were retrieved in 581 patients, with 114 of HFrEF (19.6%), 81 of HFmrEF (13.9%) and 386 of HFpEF (66.4%). The rate of comorbidities, such as hypertension, diabetes, prior transient ischemic attack (TIA)/stroke, peripheral arterial disease (PAD), chronic kidney disease (CKD) and HF, increased with the increasing CHA_2_DS_2_-VASc score. In terms of treatment, diuretics, calcium channel blockers (CCBs) and renin angiotensin system inhibitors (RASI) were used more frequently in elderly patients with AF and CAD with a higher CHA_2_DS_2_-VASc score (Table 1). The application of oral anticoagulants (OACs) and oral antiplatelets (aspirin and P2Y_12_ inhibitors) in all patients were 27.1% and 83.3%, respectively. With the increase of CHA_2_DS_2_-VASc score, no significant difference was found for the proportion of patients administrated with OACs (CHA_2_DS_2_-VASc score ≤2: 27.1%, score = 3: 28.4%, score = 4: 29.2%, score = 5: 27.3%, and score ≥6: 23.4%, *P* = 0.475) or oral antiplatelet (score ≤2: 85.9%, score = 3: 81.3%, score = 4: 81.8%, score = 5: 82.8%, and score ≥6: 85.7%, *P* = 0.404).

**Table 1:** Baseline characteristics of elderly patients with CAD and AF according to the CHA_2_DS_2_-VASc score.

**Characteristics**	**CHA_**2**_DS_**2**_-VASc ≤2** **(*n* = 192)**	**CHA_**2**_DS_**2**_-VASc = 3** **(*n* = 327)**	**CHA_**2**_DS_**2**_-VASc = 4** **(*n* = 384)**	**CHA_**2**_DS_**2**_-VASc = 5** **(*n* = 326)**	**CHA_**2**_DS_**2**_-VASc ≥6** **(*n* = 350)**	***P*** **-value**
**Demographics**						
Age, yrs, median (IQR)	72 (67–78)	76 (71–81)	77 (73–83)	79 (75–84)	80 (77–84)	<0.001
Male, *n* (%)	179 (90.2)	231 (70.6)	204 (53.1)	137 (42.0)	145 (41.4)	<0.001
BMI, kg/m^2^, median (IQR)	24 (22–27)	25 (23–27)	25 (23–27)	25 (22–27)	24 (22–27)	0.143
**Medical history**, ***n*** **(%)**						
Hypertension	65 (33.9)	239 (73.1)	289 (75.3)	273 (83.7)	318 (90.9)	<0.001
Diabetes	4 (2.1)	47 (14.4)	106 (27.6)	131 (40.2)	195 (55.7)	<0.001
Previous myocardial infarction	2 (1.0)	23 (7.0)	47 (12.2)	54 (16.6)	71 (20.3)	<0.001
Prior TIA/stroke	0 (0.0)	5 (1.5)	51 (13.3)	103 (31.6)	276 (78.9)	<0.001
Peripheral arterial disease	3 (1.6)	32 (9.8)	73 (19.0)	88 (27.0)	134(38.3)	<0.001
COPD	6 (3.1)	10 (3.1)	21 (5.5)	11 (3.4)	10 (2.9)	0.314
Hyperlipidemia	46 (24.0)	62 (19.0)	74 (19.3)	73 (22.4)	90 (25.7)	0.152
Chronic kidney disease	6 (3.1)	26 (8.0)	44 (11.5)	42 (12.9)	47 (13.4)	0.001
Liver disease	16 (8.3)	31 (9.5)	35 (9.1)	23 (7.1)	31 (8.9)	0.829
Malignancy	24 (12.5)	30 (9.2)	49 (12.8)	41 (12.6)	50 (14.3)	0.361
**Clinical presentation**, ***n*** **(%)**						
SCAD	104 (54.2)	188 (57.5)	217 (56.5)	171 (52.5)	177 (50.6)	0.342
ACS	88 (45.8)	139 (42.5)	167 (43.5)	155 (47.5)	173 (49.4)	0.342
Unstable angina	87 (45.3)	130 (39.8)	144 (37.5)	125 (38.3)	121 (34.6)	0.170
Acute myocardial infarction	1 (0.5)	11 (3.4)	27 (7.0)	31 (9.5)	54 (15.4)	<0.001
Heart failure	15 (7.8)	54 (16.5)	152 (39.6)	159 (48.8)	222 (63.4)	<0.001
**In-hospital treatment**, ***n*** **(%)**						
Diuretic	56 (29.2)	150 (45.9)	230 (59.9)	217 (66.6)	264 (75.4)	<0.001
Statins	162 (84.4)	275 (84.1)	315 (82.0)	276 (84.7)	297 (84.9)	0.842
CCB	57 (29.7)	192 (58.7)	206 (53.6)	187 (57.4)	226 (64.6)	<0.001
β-blockers	147 (76.6)	246 (75.2)	298 (77.6)	263 (80.7)	272 (77.7)	0.566
RASI	56 (29.2)	187 (57.2)	218 (56.8)	207 (63.5)	232 (66.3)	<0.001
**Antiplatelet therapy**						
Aspirin	162 (84.4)	249 (76.1)	289 (75.3)	242 (74.8)	259 (74.0)	0.074
P2Y_12_ receptor inhibitors	126 (65.6)	198 (60.6)	237 (61.7)	191 (58.6)	224 (64.0)	0.471
**Anticoagulation**						
Warfarin	26 (13.5)	59 (18.0)	68 (17.7)	59 (18.1)	54 (15.4)	0.575
NOACs	28 (14.6)	42 (12.8)	46 (12.0)	40 (12.3)	29 (8.3)	0.197
Amiodarone	64 (33.3)	96 (29.4)	83 (21.6)	79 (24.2)	98 (28.0)	0.740
PCI with drug-eluting stent	24 (12.5)	36 (11.0)	50 (13.0)	30 (9.2)	30 (8.6)	0.264

*Data are presented as the median (IQR), n (%), or n/N (%). CAD, Coronary artery disease; AF, Atrial fibrillation; BMI, Body mass index; TIA, Transient ischemic attack; COPD, Chronic obstructive pulmonary disease; SCAD, Stable coronary artery disease; ACS, Acute coronary syndrome; CCB, Calcium channel blocker; RASI, Renin angiotensin system inhibitors; NOACs, Nonvitamin K antagonist oral anticoagulants; PCI, Percutaneous coronary intervention*.

### Association Between CHA_2_DS_2_-VASc Score and Mortality

**Figure 2:**
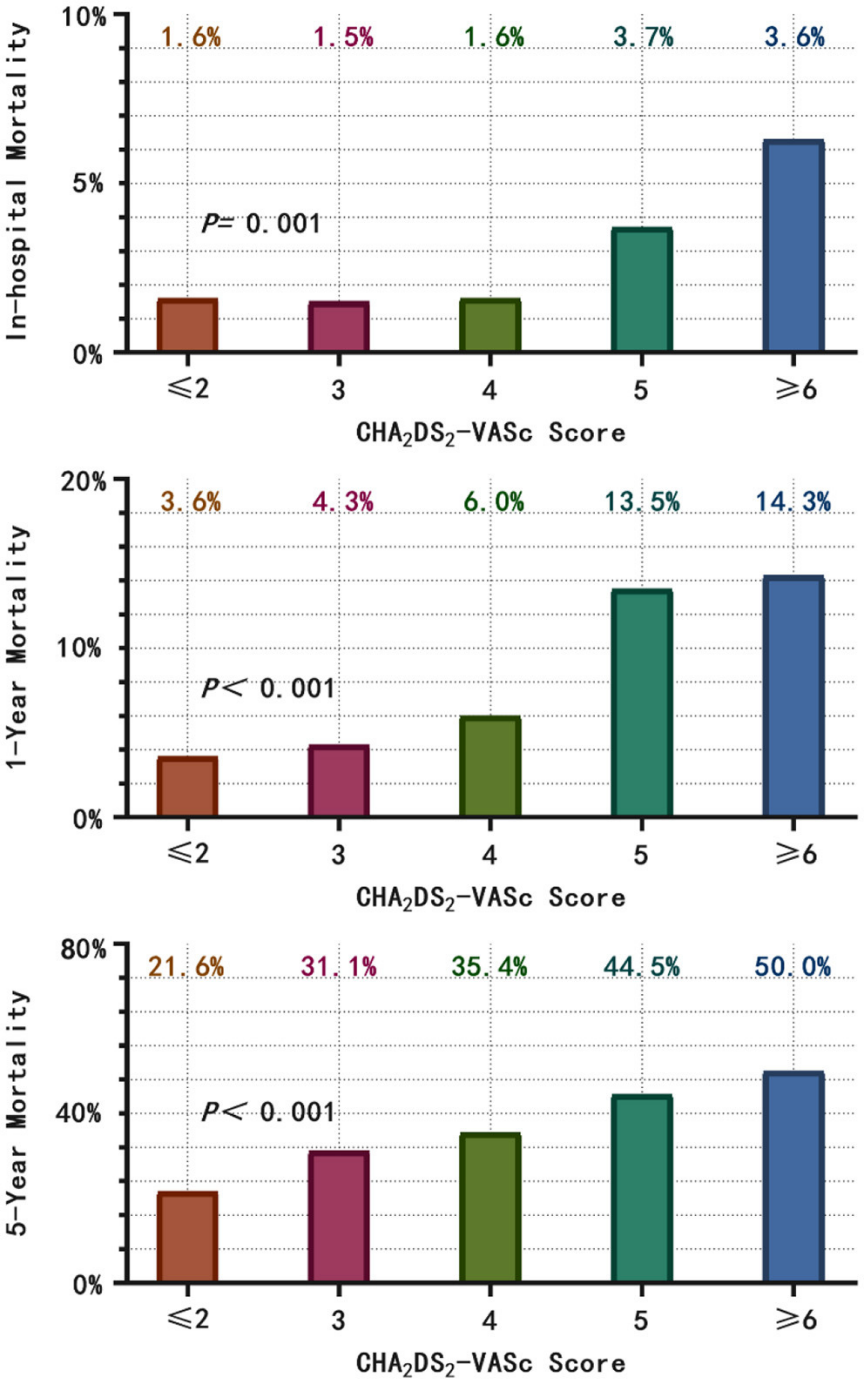
According to the CHA_2_DS_2_-VASc score category, the in-hospital, 1- and 5-year all-cause mortality were calculated. With the increase in CHA_2_DS_2_-VASc score, the in-hospital, 1- and 5-year all-cause mortality increased.

**Figure 3:**
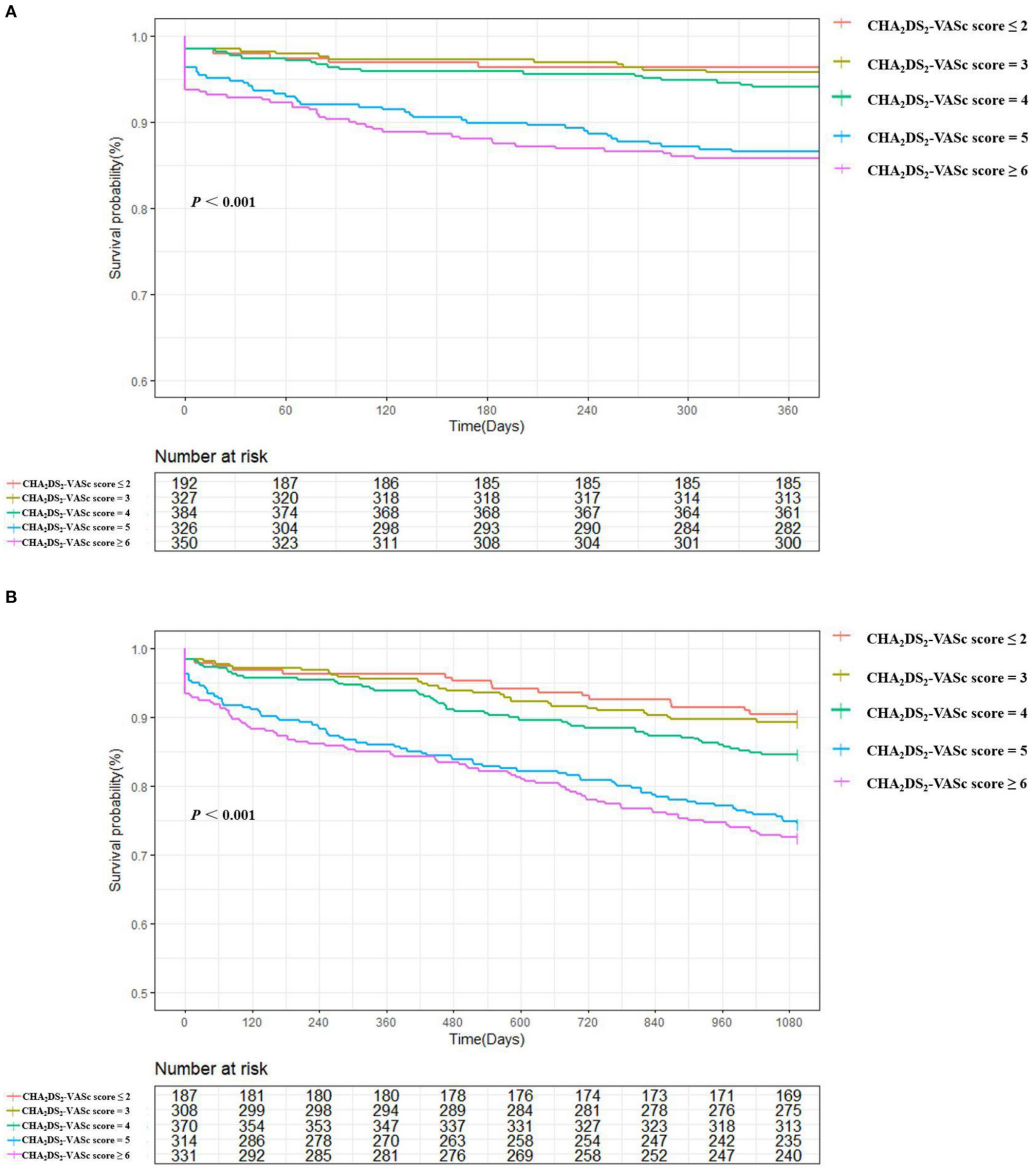
1-year **(A)** and 5-year **(B)** survival in based on Kaplan–Meier survival analysis according to CHA_2_DS_2_-VASc score: ≤2, =3, =4, =5, and ≥6. Increasing CHA_2_DS_2_-VASc scores were directly associated with reduced survival (by Log Rank Test).

A total of 582 patients died, with a mortality of 6.86 per 100 patient-years. The causes of death were cardiovascular in 152 patients (26.1%), non-cardiovascular in 247 patients (42.5%) and undetermined in 183 patients (31.4%). The in-hospital, 1-year and 5-year all-cause mortality were 3.0%, 8.7%, and 24.4%, respectively, and higher CHA_2_DS_2_-VASc scores were associated with a significantly higher mortality (Figure 2). The survival rate according to Kaplan–Meier analysis suggested that with the increase in the CHA_2_DS_2_-VASc score, the 1-year and 5-year survival rates decreased (96.4% vs. 95.7% vs. 94.0% vs. 86.5% vs. 85.7%, respectively, *P* < 0.001; 78.4% vs. 68.9% vs. 64.6% vs. 55.5% vs. 50.0%, respectively, *P* < 0.001) (Figure 3).

Compared with the patients with CHA_2_DS_2_-VASc scores <5, the patients with CHA_2_DS_2_-VASc scores ≥5 had higher risk of death (HR: 2.01, 95% CI: 1.65–2.45, *P* < 0.001). Multivariable Cox regression analysis demonstrated that CHA_2_DS_2_-VASc score ≥5 could independently predict mortality with the adjustment of the risk variables not included in the CHA_2_DS_2_-VASc score (adjusted HR: 1.78, 95% CI: 1.45–2.18, *P* < 0.001) (Table 2). Moreover, all types of HF could significantly predict the risk of death, with HFrEF contributing the most (adjusted HR for HFrEF: 2.06, 95%CI: 1.52–2.79, *P* < 0.001); for HFmrEF: 1.74, 95% CI: 1.24–2.45, *P* = 0.001; for HFpEF: 1.38, 95% CI: 1.13–1.69, *P* = 0.002, respectively).

**Table 2:** Predictors of mortality in elderly patients with CAD and AF by Cox regression analysis.

	**Univariate analysis**			^**#**^ **Multivariate analysis**		
**Variables**	**HR**	**95% CI**	***P***	**HR**	**95% CI**	***P***
Age	1.09	1.07–1.10	<0.001	1.08	1.06–1.09	<0.001
^*^BMI <18.5	1.84	1.34–2.51	<0.001	1.72	1.25–2.35	0.001
^*^BMI≥30	0.89	0.63–1.24	0.486	1.01	0.73–1.41	0.936
Previous AMI	1.62	1.31–1.99	<0.001	1.33	1.08–1.65	0.008
AMI	2.44	1.90–3.13	<0.001	2.08	1.62–2.67	<0.001
HF	2.30	1.95–2.71	<0.001	1.42	1.18–1.72	<0.001
HFrEF	2.29	1.78–2.94	<0.001	2.06	1.52–2.79	<0.001
HFmrEF	2.13	1.57–2.89	<0.001	1.74	1.24–2.45	0.001
HFpEF	1.54	1.29–1.84	<0.001	1.38	1.13–1.69	0.002
Diabetes	1.27	1.07–1.51	0.006	1.11	0.92–1.33	0.296
Prior TIA/stroke	1.28	1.08–1.52	0.005	1.05	0.90–1.14	0.487
PAD	1.90	1.59–2.64	<0.001	1.03	0.81–1.28	0.351
CKD	2.03	1.63–2.54	<0.001	2.04	1.42–2.94	<0.001
COPD	1.87	1.30–2.69	0.001	1.78	1.43–2.23	<0.001
Malignancy	1.69	1.36–2.09	<0.001	1.49	1.20–1.85	<0.001
CHA_2_DS_2_-VASc Score <5	1.0 (Reference)	1.0 (Reference)				
CHA_2_DS_2_-VASc Score ≥5	2.01	1.65–2.45	<0.001	^†^1.78	1.45–2.18	<0.001

* *Compared to the reference value of BMI 22 - <25 kg/m^2^*.

# *Adjusted by the risk factors with a statistically significant P-value <0.05 after the univariate analysis*.

† *Adjusted by the risk factors not included in the CHA_2_DS_2_-VASc score with a statistically significant P value <0.05 after the univariate analysis. CI, Confidence interval; BMI, Body mass index; AMI, Acute myocardial infarction; HF, Heart failure; HFrEF, Heart failure with reduced ejection fraction; HFmrEF, Heart failure with mid-range ejection fraction; HFpEF, Heart failure with preserved ejection fraction; AF, Atrial fibrillation; TIA, Transient ischemic attack; PAD, Peripheral arterial disease; CKD, Chronic kidney disease; COPD, Chronic obstructive pulmonary disease*.

### Prediction of the CHA_2_DS_2_-VASc Score for Mortality

In elderly patients with CAD and AF, CHA_2_DS_2_-VASc score ≥5 had a modest predictive ability for all-cause death in-hospital (C-index = 0.66, 95% CI: 0.62–0.69, *P* < 0.001), during 1-year (C-index = 0.65, 95% CI: 0.63–0.67, *P* < 0.001), and 5-year (C-index = 0.60, 95% CI: 0.59–0.61, *P* < 0.001) follow-ups. The diagnostic statistics for the CHA_2_DS_2_-VASc score of in-hospital, 1-year death, 5-year death and all death are displayed in Table 3. We also performed the internal validation by dividing the patients into those with SCAD and those with ACS. The performance of the CHA_2_DS_2_-VASc score ≥5 was comparable between the patients with SCAD and ACS for predicting in-hospital death, 1-year or 5-year death, with a moderate higher C-index in ACS patients (Table 3).

**Table 3:** Statistics of the CHA_2_DS_2_-VASc Score for the prediction of death.

	**CHA**_**2**_ **DS**_**2**_ **-VASc score ≥ 5**					
	**HL-p**	**C-index (95% CI)**	**Sensitivity (95% CI)**	**Specificity (95% CI)**	**PPV (95% CI)**	**NPV (95% CI)**
All death	0.28	0.60 (0.59–0.61)	52.6 (48.4–56.7)	67.1 (64.1–70.0)	48.3 (44.3–52.2)	70.8 (67.8–73.7)
In SCAD	0.86	0.59 (0.57–0.60)	48.2 (42.4–54.0)	68.5 (64.4–72.3)	45.0 (39.5–50.6)	71.1 (67.1–74.9)
In ACS	0.94	0.61 (0.60–0.63)	57.2 (51.2–63.0)	65.4 (60.7–70.0)	51.6 (45.9–57.2)	70.3 (65.6–74.7)
In hospital death	0.57	0.66 (0.62–0.69)	70.8 (55.7–82.6)	58.1 (55.5–60.5)	5.0 (3.6–7.0)	98.4 (97.3–99.1)
In SCAD	0.65	0.63 (0.58–0.68)	62.5 (40.8–80.4)	63.4 (60.0–66.6)	4.7 (2.7–7.8)	98.3 (96.7–99.2)
In ACS	0.93	0.68 (0.64–0.73)	79.2 (57.3–92.1)	57.7 (54.0–61.4)	6.1 (3.8–9.4)	98.8 (97.0–99.5)
1-year death	0.97	0.65 (0.63–0.67)	68.1 (59.6–75.6)	59.6 (57.0–62.2)	13.9 (11.5–16.8)	95.1 (93.4–96.4)
In SCAD	0.57	0.62 (0.58–0.65)	59.4 (46.4–71.2)	64.4 (61.0–67.8)	11.9 (8.6–16.1)	95.2 (92.9–96.8)
In ACS	0.11	0.67 (0.64–0.70)	75.3 (63.6–84.4)	60.1 (56.2–63.9)	17.5 (13.6–22.3)	95.6 (93.0–97.3)
5-year death	0.51	0.60 (0.59–0.61)	57.0 (51.9–62.0)	60.0 (56.0–63.9)	47.4 (42.8–52.1)	68.8 (64.6–72.7)
In SCAD	0.29	0.58 (0.57–0.60)	51.1 (43.7–58.3)	66.6 (62.8–70.1)	30.3 (25.4–35.7)	82.7 (79.2–85.7)
In ACS	0.52	0.61 (0.60–0.63)	60.7 (53.5–67.5)	62.9 (58.6–67.0)	37.9 (32.6–43.5)	81.1 (76.9–84.7)

*HL-p, Hosmer–Lemeshow goodness-of-fit test p-value; C-index, Concordance index; CI, Confidence interval; SCAD, Stable coronary artery disease; ACS, Acute coronary artery disease; PPV, Positive predictive value; NPV, Negative predictive value; CAD, Coronary heart disease; AF, Atrial fibrillation*.

## Discussion

In this cohort study, the main findings were that (1) the CHA_2_DS_2_-VASc score was a significant predictor of death in elderly patients with CAD and AF, and the mortality generally increased with the increasing CHA_2_DS_2_-VASc score, exhibiting a clear dose-response relationship; (2) CHA_2_DS_2_-VASc score independently and strongly predicted the in-hospital, 1- and 5-year death in elderly patients with CAD and AF. To our knowledge, this is the first study to evaluate the predictive ability of the CHA_2_DS_2_-VASc score for death in elderly patients with CAD and AF. This study could facilitate risk stratification and improve the prevention of death associated with comorbid CAD and AF in elderly patients.

AF is the most common arrhythmia, with high incidence and prevalence, and is associated with an increased risk of all-cause death and stroke (30, 31). CAD, especially AMI, will also lead to other complications and increase the risk of death (32). Age is an obvious risk factor for patients with CAD and AF (31, 33), and elderly individuals are more likely to have coexisting CAD and AF, thus the risk of death in elderly patients with CAD and AF is higher. In addition to the assessment of thromboembolic risk in patients with AF, the CHA_2_DS_2_-VASc score has been shown to predict the adverse outcomes for other cardiovascular conditions, such as chest pain (25), ACS (21, 22), AMI (24), HF (20), pulmonary emboli (23), and ACS undergoing percutaneous coronary intervention (PCI) (26). Therefore, we believe that the CHA_2_DS_2_-VASc score is a feasible predictor of prognosis in elderly patients with CAD and AF. The sensitivity of the CHA_2_DS_2_-VASc score was higher than specificity for the prediction of death in-hospital or within 1-year. It indicated that the CHA_2_DS_2_-VASc score could effectively evaluate the mortality of elderly patients with CAD and AF in-hospital or within 1 year follow-up in the study. When the CHA_2_DS_2_-VASc score is ≥5, the probability of death within 1 year will increase significantly. Additionally, with the increase in the CHA_2_DS_2_-VASc score, the in-hospital, 1- and 5-year mortality also increased. These results indicate that the CHA_2_DS_2_-VASc score could predict the prognosis of elderly patients with CAD and AF.

The risk factors for death in elderly patients with CAD and AF were assessed for the first time in this cohort. We found that in addition to the CHA_2_DS_2_-VASc score, the independent risk factors for all-cause death in elderly patients with CAD and AF included BMI <18.5 kg/m^2^, previous or current AMI, CKD, COPD, and malignancy. Similar risk factors for death were also found in AF patients in the ROCKET-AF study (34) and the GARFIELD-AF global prospective registry (35), suggesting that overall mortality due to AF is tightly linked to the same risk factors and comorbidities. Our results also emphasized the prognostic importance of underweight (BMI <18.5 kg/m^2^) in the elderly population. We found that BMI <18.5 kg/m^2^ was a significant predictor of overall mortality, which probably reflects the known association between a decrease in BMI and an increase in mortality in CAD patients, regardless of the baseline BMI value (36). Further studies are needed to better understand the impact of the combination of risk factors on mortality in the elderly population.

The combination of AF and CAD is a common and complex clinical condition in which to address anticoagulation therapy (37), especially in elderly patients. Taking OACs can reduce the risk of embolism, but it also increases the risk of bleeding. Therefore, whether to take OACs should be judged by the patient's health situation (38). If AF develops during the first year after ACS and there is an indication for thromboembolic prevention with anticoagulation, OACs should be started. In stable CAD patients with AF, oral anticoagulation is necessary when the CHA_2_DS_2_-VASc score is ≥2 (39). Elderly patients requiring anticoagulation for AF are at higher risk of adverse outcomes, but also have a higher absolute benefit from OAC (40). However, in our study, the application rate of OACs was only 27.1%, and it did not increase with the CHA_2_DS_2_-VASc score, suggesting that the application of OACs in elderly patients with CAD and AF was not sufficient, which might attribute to the increasing risk of death in patients with higher value of CHA_2_DS_2_-VASc score. Previous studies have found that the application of OACs in the elderly population was insufficient (41). Therefore, the use of OACs in elderly patients with CAD and AF should be increased, and further studies are needed to verify whether the application of OAC could decrease the mortality in these patients.

Several limitations of this study warrant consideration. The present study was an observational real-world study in which we did not exclude severely ill patients (who are typically excluded from clinical trials), thus, the mortality in elderly patients with CAD and AF might be higher than expected in clinical trials. Data on the clinical parameters and drug therapies were obtained from electronic health record. Although the data was validated and found to be highly accurate, not all clinical characteristics could be verified. While we tried to make adjustment for the clinically relevant parameters, it is impossible to adjust for all variables that may affect the outcomes. In addition, the study was based on a single-center cohort, and the findings should be validated in large multicenter cohorts.

## Conclusion

The CHA_2_DS_2_-VASc score could independently predict all-cause death in the elderly patients with concomitant CAD and AF.

## Data Availability Statement

The original contributions presented in the study are included in the article/supplementary material, further inquiries can be directed to the corresponding author/s.

## Ethics Statement

The studies involving human participants were reviewed and approved by Institutional Ethics Committee of the General Hospital of the People's Liberation Army. The patients/participants provided their written informed consent to participate in this study.

## Author Contributions

TY, JM, and YZha: study concept and design. YW, JL, LD, LQ, HY, WG, XF, GW, ZW, RD, and YZou: acquisition of data. GW, YW, LQ, LD, and TY: analysis and interpretation of data. GW, YW, LD, LQ, and TY: drafting of the manuscript. TY and JM: critical revision of the manuscript for important intellectual content. All authors contributed to the article and approved the submitted version.

## Funding

This work was supported by grants from the National Natural Science Foundation of China (Nos. 81870262 and 82170352).

## Conflict of Interest

The authors declare that the research was conducted in the absence of any commercial or financial relationships that could be construed as a potential conflict of interest.

## Publisher's Note

All claims expressed in this article are solely those of the authors and do not necessarily represent those of their affiliated organizations, or those of the publisher, the editors and the reviewers. Any product that may be evaluated in this article, or claim that may be made by its manufacturer, is not guaranteed or endorsed by the publisher.

## References

[1] Michniewicz E Mlodawska E Lopatowska P Tomaszuk-Kazberuk A Malyszko J. Patients with atrial fibrillation and coronary artery disease - double trouble. Adv Med Sci. (2018) 63:30–5. 10.1016/j.advms.2017.06.005 28818746

[2] Madhavan MV Gersh BJ Alexander KP Granger CB Stone GW. coronary artery disease in patients >/=80 years of age. J Am Coll Cardiol. (2018) 71:2015–40. 10.1016/j.jacc.2017.12.068 29724356

[3] Hindricks G Potpara T Dagres N Arbelo E Bax JJ Blomstrom-Lundqvist C. 2020 ESC Guidelines for the diagnosis management of atrial fibrillation developed in collaboration with the European association for cardio-thoracic surgery (EACTS): the task force for the diagnosis management of atrial fibrillation of the European society of cardiology (ESC) developed with the special contribution of the european heart rhythm association (EHRA) of the ESC. Eur Heart J. (2021) 42:373–98. 10.1093/eurheartj/ehaa612 34520521

[4] Wang J Yang YM Zhu J. Mechanisms of new-onset atrial fibrillation complicating acute coronary syndrome. Herz. (2015) 1(Suppl. 40):18–26. 10.1007/s00059-014-4149-3 25352243

[5] Benjamin EJ Blaha MJ Chiuve SE Cushman M Das SR Deo R. Heart disease and stroke statistics-2017 update: a report from the american heart association. Circulation. (2017) 135:e146–603. 10.1161/CIR.0000000000000485 28122885 PMC5408160

[6] Fihn SD Gardin JM Abrams J Berra K Blankenship JC Dallas AP. 2012 ACCF/AHA/ACP/AATS/PCNA/SCAI/STS guideline for the diagnosis and management of patients with stable ischemic heart disease: a report of the American college of cardiology foundation/American heart association task force on practice guidelines, and the American college of physicians, American association for thoracic surgery, preventive cardiovascular nurses association, society for cardiovascular angiography and interventions, and society of thoracic surgeons. J Am Coll Cardiol. (2012) 60:e44–164. 10.1016/j.jacc.2012.07.013 23182125

[7] Fihn SD Blankenship JC Alexander KP Bittl JA Byrne JG Fletcher BJ. 2014 ACC/AHA/AATS/PCNA/SCAI/STS focused update of the guideline for the diagnosis and management of patients with stable ischemic heart disease: a report of the American college of cardiology/American heart association task force on practice guidelines, and the American association for thoracic surgery, preventive cardiovascular nurses association, society for cardiovascular angiography and interventions, and society of thoracic surgeons. J Am Coll Cardiol. (2014) 64:1929–49. 10.1016/j.jacc.2014.07.017 25077860

[8] Dai X Busby-Whitehead J Forman DE Alexander KP. Stable ischemic heart disease in the older adults. J Geriatr Cardiol. (2016) 13:109–14. 10.11909/j.issn.1671-5411.2016.02.013 27168734 PMC4854947

[9] Tegn N Abdelnoor M Aaberge L Endresen K Smith P Aakhus S. Invasive versus conservative strategy in patients aged 80 years or older with non-ST-elevation myocardial infarction or unstable angina pectoris (After Eighty study): an open-label randomised controlled trial. Lancet. (2016) 387:1057–65. 10.1016/S0140-6736(15)01166-6 26794722

[10] Pedersen OD Bagger H Kober L Torp-Pedersen C. The occurrence and prognostic significance of atrial fibrillation/-flutter following acute myocardial infarction. TRACE study group. TRAndolapril cardiac evalution. Eur Heart J. (1999) 20:748–54. 10.1053/euhj.1998.1352 10329066

[11] Rathore SS Berger AK Weinfurt KP Schulman KA Oetgen WJ Gersh BJ. Acute myocardial infarction complicated by atrial fibrillation in the elderly: prevalence and outcomes. Circulation. (2000) 101:969–74. 10.1161/01.CIR.101.9.969 10704162

[12] Lopes RD Pieper KS Horton JR Al-Khatib SM Newby LK Mehta RH. Short- and long-term outcomes following atrial fibrillation in patients with acute coronary syndromes with or without ST-segment elevation. Heart. (2008) 94:867–73. 10.1136/hrt.2007.134486 18332062

[13] Schmitt J Duray G Gersh BJ Hohnloser SH. Atrial fibrillation in acute myocardial infarction: a systematic review of the incidence, clinical features and prognostic implications. Eur Heart J. (2009) 30:1038–45. 10.1093/eurheartj/ehn579 19109347

[14] Chugh SS Havmoeller R Narayanan K Singh D Rienstra M Benjamin EJ. Worldwide epidemiology of atrial fibrillation: a global burden of disease 2010 study. Circulation. (2014) 129:837–47. 10.1161/CIRCULATIONAHA.113.005119 24345399 PMC4151302

[15] Kundu A O'Day K Shaikh AY Lessard DM Saczynski JS Yarzebski J. Relation of atrial fibrillation in acute myocardial infarction to in-hospital complications and early hospital readmission. Am J Cardiol. (2016) 117:1213–8. 10.1016/j.amjcard.2016.01.012 26874548 PMC5075423

[16] Wi J Shin DH Kim JS Kim BK Ko YG Choi D. Transient new-onset atrial fibrillation is associated with poor clinical outcomes in patients with acute myocardial infarction. Circ J. (2016) 80:1615–23. 10.1253/circj.CJ-15-1250 27210266

[17] Crenshaw BS Ward SR Granger CB Stebbins AL Topol EJ Califf RM. Atrial fibrillation in the setting of acute myocardial infarction: the GUSTO-I experience. Global utilization of streptokinase and TPA for occluded coronary arteries. J Am Coll Cardiol. (1997) 30:406–13. 10.1016/S0735-1097(97)00194-0 9247512

[18] Stenestrand U Lindback J Wallentin L Registry R.-H. Anticoagulation therapy in atrial fibrillation in combination with acute myocardial infarction influences long-term outcome: a prospective cohort study from the register of information and knowledge about swedish heart intensive care admissions (RIKS-HIA). Circulation. (2005) 112:3225–31. 10.1161/CIRCULATIONAHA.105.552984 16301355

[19] Mehta RH Dabbous OH Granger CB Kuznetsova P Kline-Rogers EM Anderson A Jr. Comparison of outcomes of patients with acute coronary syndromes with and without atrial fibrillation. Am J Cardiol. (2003) 92:1031–6. 10.1016/j.amjcard.2003.06.001 14583352

[20] Shuvy M Zwas DR Keren A Gotsman I. Value of the CHA2 DS2 -VASc score for predicting outcome in patients with heart failure. ESC Heart Fail. (2020) 7:2553–60. 10.1002/ehf2.12831 32614479 PMC7524134

[21] Chua SK Lo HM Chiu CZ Shyu KG. Use of CHADS(2) and CHA(2)DS(2)-VASc scores to predict subsequent myocardial infarction, stroke, and death in patients with acute coronary syndrome: data from Taiwan acute coronary syndrome full spectrum registry. PLoS One. (2014) 9:e111167. 10.1371/journal.pone.0111167 25343586 PMC4208805

[22] Rozenbaum Z Elis A Shuvy M Vorobeichik D Shlomo N Shlezinger M. CHA2DS2-VASc score and clinical outcomes of patients with acute coronary syndrome. Eur J Intern Med. (2016) 36:57–61. 10.1016/j.ejim.2016.09.010 27707608

[23] Gok M Kurtul A Harman M Kara M Suleymanoglu M Ornek E. Relationship between CHA2DS2-VASc score right ventricular dysfunction in patients with acute pulmonary thromboembolism. Clin Appl Thromb Hemost. (2018) 24(Suppl. 9):56S–62S. 10.1177/1076029618785771 29996663 PMC6714857

[24] Li CY Chang CJ Chung WJ Lin CJ Hsueh SK Lee CH. Assessment of CHA2DS2-VASc score for predicting cardiovascular and cerebrovascular outcomes in acute myocardial infarction patients. Medicine. (2018) 97:e11230. 10.1097/MD.0000000000011230 29995755 PMC6076158

[25] Topaz G Haisraely O Shacham Y Beery G Shilo L Kassem N. CHA2 DS2 -VASc score and clinical outcomes of patients with chest pain discharged from internal medicine wards following acute coronary syndrome rule-out. Clin Cardiol. (2018) 41:539–43. 10.1002/clc.22925 29687656 PMC6489928

[26] Ma X Shao Q Dong L Cheng Y Lv S Shen H. Prognostic value of CHADS2 and CHA2DS2-VASc scores for post-discharge outcomes in patients with acute coronary syndrome undergoing percutaneous coronary intervention. Medicine. (2020) 99:e21321. 10.1097/MD.0000000000021321 32791726 PMC7387006

[27] Berrington de Gonzalez A Hartge P Cerhan JR Flint AJ Hannan L MacInnis RJ. Body-mass index and mortality among 1.46 million white adults. N Engl J Med. (2010) 363:2211–9. 10.1056/NEJMoa1000367 21121834 PMC3066051

[28] European Heart Rhythm A European Association for Cardio-Thoracic S Camm AJ Kirchhof P Lip GY Schotten U. Guidelines for the management of atrial fibrillation: the task force for the management of atrial fibrillation of the european society of cardiology (ESC). Eur Heart J. (2010) 31:2369–429. 10.1093/eurheartj/ehq278 20802247

[29] January CT Wann LS Calkins H Chen LY Cigarroa JE Cleveland C Jr. 2019 AHA/ACC/HRS focused update of the 2014 AHA/ACC/HRS guideline for the management of patients with atrial fibrillation: a report of the American college of cardiology/American heart association task force on clinical practice guidelines and the heart rhythm society in collaboration with the society of thoracic surgeons. Circulation. (2019) 140:e125–51. 10.1161/CIR.0000000000000665 30686041

[30] Wilke T Groth A Mueller S Pfannkuche M Verheyen F Linder R. Incidence and prevalence of atrial fibrillation: an analysis based on 8.3 million patients. Europace. (2013) 15:486–93. 10.1093/europace/eus333 23220354

[31] Odutayo A Wong CX Hsiao AJ Hopewell S Altman DG Emdin CA. Atrial fibrillation and risks of cardiovascular disease, renal disease, and death: systematic review and meta-analysis. BMJ. (2016) 354:i4482. 10.1136/bmj.i4482 27599725

[32] Shao C Wang J Tian J Tang YD. Coronary artery disease: from mechanism to clinical practice. Adv Exp Med Biol. (2020) 1177:1–36. 10.1007/978-981-15-2517-9_1 32246442

[33] Malakar AK Choudhury D Halder B Paul P Uddin A Chakraborty S. A review on coronary artery disease, its risk factors, and therapeutics. J Cell Physiol. (2019) 234:16812–823. 10.1002/jcp.28350 30790284

[34] Pokorney SD Piccini JP Stevens SR Patel MR Pieper KS Halperin JL. Cause of death and predictors of all-cause mortality in anticoagulated patients with nonvalvular atrial fibrillation: data from ROCKET AF. J Am Heart Assoc. (2016) 5:e002197. 10.1161/JAHA.115.002197 26955859 PMC4943233

[35] Gibbs H Freedman B Rosenqvist M Virdone S Mahmeed WA Ambrosio G. Clinical outcomes in asymptomatic and symptomatic atrial fibrillation presentations in GARFIELD-AF: implications for AF screening. Am J Med. (2021) 134 893–901 e811. 10.1016/j.amjmed.2021.01.017 33607088

[36] Dong SY Yan ST Wang ML Li ZB Fang LQ Zeng Q. Associations of body weight and weight change with cardiovascular events and mortality in patients with coronary heart disease. Atherosclerosis. (2018) 274:104–11. 10.1016/j.atherosclerosis.2018.05.007 29763769

[37] Steffel J Verhamme P Potpara TS Albaladejo P Antz M Desteghe L. The 2018 European heart rhythm association practical guide on the use of non-vitamin K antagonist oral anticoagulants in patients with atrial fibrillation. Eur Heart J. (2018) 39:1330–93. 10.1093/eurheartj/ehy136 29562325

[38] Wojszel ZB. Dementia diagnoses and treatment in geriatric ward patients: a cross-sectional study in poland. Clin Interv Aging. (2020) 15:2183–94. 10.2147/CIA.S281723 33223824 PMC7671484

[39] Verheugt FWA Ten Berg JM Storey RF Cuisset T Granger CB. Antithrombotics: from aspirin to DOACs in coronary artery disease and atrial fibrillation (Part 3/5). J Am Coll Cardiol. (2019) 74:699–711. 10.1016/j.jacc.2019.02.080 31277840

[40] Bauersachs RM Herold J. Oral anticoagulation in the elderly and frail. Hamostaseologie. (2020) 40:74–83. 10.1055/s-0040-1701476 32000266

[41] Dregan A Ravindrarajah R Charlton J Ashworth M Molokhia M. Long-term trends in antithrombotic drug prescriptions among adults aged 80 years and over from primary care: a temporal trends analysis using electronic health records. Ann Epidemiol. (2018) 28:440–6. 10.1016/j.annepidem.2018.03.006 29609872

